# Clinical performance of two commercially available rapid antigen tests for influenza, RSV, and SARS-CoV-2 diagnostics

**DOI:** 10.1128/spectrum.01630-24

**Published:** 2024-11-26

**Authors:** Laura E. Savolainen, Joanna Peltola, Risto Hilla, Tapio Åman, Markku Broas, Ilkka S. Junttila

**Affiliations:** 1 NordLab, Oulu, Finland; 2 NordLab, Rovaniemi, Finland; 3 Mehiläinen Länsi-Pohja, Kemi, Finland; 4 Wellbeing Cervices County of Lapland, Rovaniemi, Finland; 5 University of Oulu, Oulu, Finland; 6 Tampere University, Tampere, Finland; 7 Fimlab Laboratories, Tampere, Finland; University of Chicago, Chicago, Illinois, USA

**Keywords:** antigen test, influenza, respiratory syncytial virus, SARS-CoV-2

## Abstract

Rapid antigen tests (RATs) might provide rapid, low-cost, and easy-to-perform choice to nucleic-acid amplification methods (NAAT) in point-of-care (POC) diagnostics of respiratory infections. The clinical performance of two commercial combo-RATs for influenza A and B, respiratory syncytial virus (RSV) and severe acute respiratory syndrome coronavirus 2 (SARS-CoV-2) POC diagnostics, was assessed. RAT and real-time-PCR (RT-PCR) analysis were performed on 620 patients admitted to the emergency department due to respiratory symptoms. The sensitivity and specificity of the RATs compared with RT-PCR results were studied. The sensitivity of the RATs with respect to symptom duration was also estimated. The tested RAT1 showed sensitivities of 98.68%, 73,53%, and 44.44% for SARS-CoV-2, influenza A, and RSV, respectively, when the cycle threshold (Ct) values were <25 and 95.45%, 68.42%, 40.0% with Ct values <30. In RAT2 analysis, respective sensitivities for influenza A and RSV in samples with Ct <25 were 85% and 100% and in samples with Ct <30 respective sensitivities dropped to 69.23% and 66.67%. Positive SARS-CoV-2 cases during the RAT2 testing period and influenza B cases during both the RAT1 and RAT2 testing periods were scarce, so they were omitted from the statistical analysis. The specificity of both tested RATs was high and varied between 99.48% and 100%. The clinical use of RATs should be carefully considered due to the possibility of insufficient sensitivity. Because the specificity of the tests is high, the use of such tests might provide added value, for example, in emergency units, particularly when the prevalence of these infections is high. However, an option to confirm negative RAT by NAAT should be available.

**IMPORTANCE:**

Rapid diagnostics of respiratory viruses is essential for clinical management, infection control, and epidemiological surveillance. Our results showed that due to the high specificity and still inadequate sensitivity, rapid antigen tests (RATs) might be useful for influenza, respiratory syncytial virus, and severe acute respiratory syndrome coronavirus 2 point-of-care diagnostics when correctly used. The results provide useful information for laboratory and infectious disease control professionals to support decision-making when implementation of the RATs in clinical use is planned.

## INTRODUCTION

Acute respiratory tract infections caused by influenza A (Inf A), influenza B (Inf B), respiratory syncytial virus (RSV), and severe acute respiratory syndrome coronavirus 2 (SARS-CoV-2) are indistinguishable purely by clinical appearance. In addition, there are a plethora of viruses that may cause similar symptoms. Hence, rapid diagnostics of these viruses is essential not only for clinical management but also for infection control and epidemiological surveillance. Rapid antigen tests (RATs) might offer lower costs and easy-to-perform choice compared with nucleic-acid amplification methods (NAATs), such as real-time-PCR (RT-PCR) in point-of-care (POC) facilities. Previously, the sensitivity of RATs was shown to be lower than NAATs, especially in samples with low viral loads (1–3). A relatively high variation in clinical performance between different commercial RATs has also been reported (4, 5). While viral culture from the patient sample remains the golden standard method for detecting an active-replicative virus, it is rarely feasible due to the relatively slow turnaround time in the laboratory (6).

In this pilot study, the clinical performance of two commercial combo RATs, SARS-CoV-2/Inf A + B/RSV Antigen Combo Rapid Test (Alltest) (in this report, RAT1) and SARS-CoV-2/Flu A + B/RSV Antigen Rapid Test (Qingdao HighTop) (in this report RAT2), was independently evaluated by comparing the test results with commercial RT-PCR. The study was implemented in two central hospital emergency units located in Northern Finland during the Inf A, RSV, and SARS-COV-2 epidemic season 2023–2024.

## RESULTS

### Clinical performance of RAT1

A total of 420 samples were analyzed with RAT1 and 15 samples were excluded from the statistical analysis. The exclusion was due to either the lack of RT-PCR result (*n* = 11) for the corresponding RAT1 result or unclear RAT result (*n* = 4). The results obtained with RAT1 are shown in Table 1.

**TABLE 1:** Clinical performance of RAT1 in RT-PCR-positive samples with different Ct values

	RT-PCR-positive (%)	Sensitivity (95% CI)	Specificity (95% CI)	PPV (95% CI)	NPV (95% CI)	Accuracy (95% CI)
SARS-CoV-2, Ct any^*a*^	25.68	82.69 (74.03–89.41)	100 (98.78–100)	100 (95.80–100)	94.36 (91.66–96.22)	95.56 (93.07–97.34)
SARS-CoV-2, Ct <30	21.73	95.45 (88.77–98.75)	100 (98.84–100)	100 (95.70–100)	98.75 (96.85–99.52)	99.01 (97.49–99.73)
SARS-CoV-2, Ct <25	18.77	98.68 (92.89–99.97)	100 (98.89–100)	100 (95.20–100)	99.70 (97.91–99.96)	99.75 (98.63–99.99)
Inf A, Ct any^*a*^	11.36	56.52 (41.11–71.07)	100 (98.98–100)	100 (86.77–100)	94.72 (92.81–96.15)	95.06 (92.48–96.96)
Inf A, Ct <30	9.38	68.42 (51.35–82.50)	100 (99.00–100)	100 (86.77–100)	96.83 (95.04–97.99)	97.04 (94.88–98.46)
Inf A, Ct <25	8.40	73.53 (55.64–87.12)	100 (99.01–100)	100 (86.28–100)	97.63 (95.93–98.63)	97.78 (95.82–98.98)
RSV, Ct any^*a*^	2.47	40.00 (12.16–73.76)	99.75 (98.60–99.99)	80.00 (32.89–97.03)	98.50 (97.54–99.09)	98.27 (96.47–99.30)
RSV, Ct <30	2.47	40.00 (12.16–73.76)	99.75 (98.60–99.99)	80.00 (32.89–97.03)	98.50 (97.54–99.09)	98.27 (96.47–99.30)
RSV, Ct <25	2.22	44.44 (13.70–78.80)	99.75 (98.60–99.99)	80.00 (33.11–97.00)	98.75 (97.78–99.30)	98.52 (96.80–99.45)

^*a*^ Interpreted RT-PCR positive according to the manufacturer.

As expected, the highest sensitivities were observed for samples with higher viral loads (i.e., cycle threshold [Ct] <25) for all tested viruses. However, a high variation in sensitivity between tested viruses was observed. With SARS-CoV-2, Inf A and RSV RAT1 achieved sensitivities of 98.68%, 73,53%, and 44.44%, respectively, when the Ct values were <25 and 95.45%, 68.42%, and 40.0% with Ct values <30.

The total specificity of all individual test results for SARS-CoV-2, Inf A, Inf B, and RSV (*n* = 1620) was 99.9%, indicating that if the test is positive, then the positive result is most likely reliable. All the samples that were negative for SARS-CoV-2 and Inf A in RT-PCR were also negative in RAT for these viruses. One sample gave RSV-positive result in RAT but was negative in RT-PCR. In the same sample, Inf A was simultaneously recognized by both methods. Altogether 3/10 RSV RT-PCR-positive samples were positive also for Inf A or SARS-CoV-2. All of these samples were negative with RSV RAT regardless of the RT-PCR Ct value.

Unfortunately, there were no Inf B RT-PCR-positive samples in the material during the study period for RAT1. In one sample, RAT1 was interpreted positive for Inf B, but RT-PCR for this sample was negative. This result was not further analyzed.

### Clinical performance of RAT2

Altogether 206 samples were analyzed and 2 samples were excluded from the statistical analysis due to unclear RAT results. There were only five SARS-CoV-2 RT-PCR-positive samples in the study period for RAT2. In 4/5 of these samples, the Ct values were higher than 30 and these samples were negative in RAT2. One sample with SARS-CoV-2 Ct value <25 was positive also in RAT2. The same sample was also Inf A RT-PCR positive (Ct <25) but Inf A negative in RAT2.

As in RAT1, the sensitivity on RAT2 was higher when RT-PCR Ct values were <25 compared with higher Ct values (Table 2). The respective sensitivities for Inf A and RSV in samples with Ct <25 were 85% and 100%. The sensitivity fell to 69.23% for Inf A and 66.67% for RSV when the RAT2 results were compared with the RT-PCR sample with Ct value <30.

**TABLE 2:** Clinical performance of RAT2 in RT-PCR-positive samples with different Ct values

	RT-PCR-positive (%)	Sensitivity ( 95% CI)	Specificity (95% CI)	PPV (95% CI)	NPV (95% CI)	Accuracy (95% CI)
Inf A, Ct any^*a*^	17.65	50.00 (32.92–67.08)	100 (97.83–100)	100 (81.47–100)	90.32 (87.07–92.83)	91.18 (86.41–94.69)
Inf A, Ct <30	12.75	69.23 (48.21–85.67)	100 (97.95–100)	100 (81.47–100)	95.70 (92.59–97.54)	96.08 (94.42–98.29)
Inf A, Ct <25	9.80	85.00 (62.11–96.79)	100 (98.02–100)	100 (80.49–100)	98.40 (95.58–99.43)	98.53 (95.76–99.70)
RSV, Ct any^*a*^	5.39	54.55 (23.38–83.25)	99.48 (97.15–99.99)	85.71 (44.12–97.85)	97.46 (95.26–98.66)	97.06 (93.71–98.91)
RSV, Ct <30	4.41	66.67 (29.93–92.51)	99.49 (97.18–99.99)	85.71 (44.59–97.81)	98.48 (96.25–99.39)	98.04 (95.06–99.46)
RSV, Ct <25	2.45	100 (47.82–100)	99.50 (97.23–99.99)	83.33 (41.44–97.25)	100 (98.15–100)	99.51 (97.30–99.99)

^*a*^ Interpreted RT-PCR positive according to the manufacturer.

Total specificity was 99.75% when all individual test results for SARS-CoV-2, Inf A, Inf B, and RSV (*n* = 816) were analyzed. In one sample, Inf A was detected by RT-PCR, but RAT2 identified Inf B, and in one sample RT-PCR Inf A-positive sample was interpreted as RSV by RAT2. These results were not further analyzed.

### Effects of the duration of the symptoms for RAT1 and RAT2 positivity

To understand whether RAT positivity correlated with symptom duration, the RAT1 and RAT2 results in RT-PCR-positive samples were compared with self-reported symptom duration. The mean of the symptom duration (in days) appeared higher in RAT-negative patients compared with RAT-positive patients for all analyzed viruses and RATs (Table 3). However, this difference was not statistically significant, which may be in part due to the relatively small sample groups. The difference was the highest in Inf A RT-PCR-positive (Ct any or Ct <30) patients and statistically significant (*P* <0.5) when the sample size was increased by pooling results of both RATs (Table 4).

**TABLE 3:** The difference of symptoms duration in RAT-positive and -negative patients in RT-PCR positive (Ct any; interpreted RT-PCR positive according to the manufacturer) population

		RAT1				RAT2			
		*n*	Mean (days)	Mean difference (95% CI)	*P* value	*n*	Mean (days)	95% CI	*P* value
SARS-CoV-2	RAT positive	63	3.2	0.4 (–2.8 to 2.1)	0.7710				
	RAT negative	15	3.6						
Inf A	RAT positive	23	3.0	1.9 (–4.9 to 1.0)	0.1936	13	2.8	2.7 (–5.5 to 0.2)	0.0629
	RAT negative	18	4.9			12	5.5		
RSV	RAT positive	4	3.8	0.4 (–4.3 to 3.5)	0.8127	4	3.0	1.0 (–8.0 to 6.0)	0.7123
	RAT negative	6	4.2			2	4.0		

**TABLE 4:** The difference of symptoms duration in RAT-positive and -negative patients in RT-PCR-positive population when RAT1 and RAT2 results were pooled

		*n*	Mean (days)	Mean difference (95% CI)	*P* value
Pooled RAT1 and RAT2, Ct any**^*a*^**					
Inf A	RAT positive	36	2.9	2.2 (−4.3 to −0.2)	0.0353^*a*^
	RAT negative	30	5.2		
RSV	RAT positive	8	3.4	0.8 (−3.5 to 2)	0.5651
	RAT negative	8	4.1		
Pooled RAT1 and RAT2, Ct <30					
Inf A	RAT positive	35	2.9	2.6 (-5.2 to −0.1)	0.0452^*a*^
	RAT negative	17	5.5		

^*a*^ Interpreted RT-PCR positive according to the manufacturer.

## DISCUSSION

POC testing facilitates the decision-making concerning patient isolation, possible antiviral treatment initiation, or infection control, particularly in the emergency department (ED). In addition, the benefits become more clear in areas with long distances to central laboratories and units with a moderate number of patients. For example, in the NordLab catchment area in Northern Finland, many elderly care units with relatively low numbers of inhabitants have 100–200 km distance to the nearest central laboratory running RT-PCR diagnostics and would greatly benefit from sensitive and specific POC tests. The maintenance costs of POC-PCR instruments would be high and increase the costs per sample, especially in seasonal infections. Therefore, lateral-flow RATs would be a suitable alternative.

The accuracy of the variety of commercial SARS-CoV-2 RATs was recently comprehensively explored in a Cochrane study (4). Sensitivities of the RATs varied from 34.4% to 91.3% when symptomatic patients were tested. In contrast, specificity was found to be high, 97–100%, in most of the tests in which specificity was reported. The accuracy of the RATs is dependent on the tested population and chosen sample material, for example, nasal, nasopharyngeal, oropharyngeal, and throat swabs are used. Frank et al. reported lower sensitivity in RSV RATs when throat swab was used instead of nasal, nasopharyngeal, or trachea secretes (7). No significant difference between nasal and nasopharyngeal swab was reported in the study comparing SARS-CoV-2 and RSV RAT (8). In our study, only nasopharyngeal swabs were analyzed.

When clinical performance comparisons between NAAT and RAT are done, the viral load is also an important question. For example, sensitivities of 100%, 98.25%, and 88.64% were reported in SARS-CoV-2 RAT with respective Ct values of 20, 25, and 30 (9). Jang et al. evaluated three commercial RSV RATs and reported negative RAT results in two of the tests when the RT-PCR Ct values were increased by more than 25 (10). These results are quite similar to the results of our study and a Belgium study regarding another commercial fourplex RAT (11). In fact, Bryant et al. reported sensitivity drop for Inf A from 87.1% to 57.1% when the sample Ct values were increased from <25 to <30.

Virus culture remains the golden standard in estimating how contagious the virus infection is. The viability of SARS-CoV-2 in culture compared with NAAT Ct values was reviewed by two recent studies (11, 12). Both studies concluded that a viable virus was still found when Ct values were approximately 35. However, when the Ct values were increased by more than 30, viable virus was detected only in a minority of the cases. Furthermore, results in which viable SARS-CoV2 virus was no longer detected when the Ct values were increased by more than 25 have also been reported (13).

In this study, the clinical performance of two commercial four-plex combo RATs for POC testing of symptomatic patients at the hospital emergency unit was evaluated. The sensitivity of the tests was higher for SARS-CoV-2 than for Inf A or RSV. Furthermore, the sensitivity of RATs was higher in samples with lower Ct values, revealing higher viral load correlates with better performance of RAT compared with the samples with higher Ct values. This result was shown in all analyzed viruses, SARS-CoV-2, Inf A, and RSV. The specificity of the tests was high and varied between 99.48% and 100%.

Because only moderate sensitivity for Inf A and RSV was observed, the clinical use of the tests needs to be carefully assessed. Also, for SARS-CoV-2, the sensitivity was lower by RAT than RT-PCR, especially when the Ct values were more than 30. Still, these tested RATs might have added value in improving patient cohortation in emergency units and in primary health care units with long distances to the central laboratory. A positive RAT result might help when making decisions on patients’ isolation or antiviral treatment initiation. However, when RAT is negative, confirmatory NAAT should be done.

Accuracy of the SARS-CoV-2, influenza, and RSV RATs has been shown to be the highest during the few days after the onset of symptoms (3, 9, 14). In NAATs, the positive results occur even weeks after the onset of the infection (9). Montano et al. reported that viable virus was no longer detectable in the samples collected after 10 days of symptom onset (13). Similar results were also reported in a recent review article by Walsh and co-workers (11). When organ transplant patients with immunosuppressive treatment protocols were studied, viable viruses were still detectable after 150 days from the onset of infection (12).

Our results are in line with the findings that show that the length of symptoms would correlate with RAT positivity in RT-PCR-positive patients. For example, in Inf A RT-PCR-positive patients, the mean of the days of symptom duration during the sampling time was significantly higher in the RAT-negative than in the RAT-positive group. However, the duration of the symptoms was quite short (1–5 days) in our study population in most of the patients, but this is most often the case in acute upper respiratory tract infections. However, there were a few cases in which symptom duration was 2 weeks or longer. Interestingly, few positive RAT and RT-PCR results were found also in this group. Symptom information was collected only as reported by the patient, which may somewhat compromise these results as medical records were not analyzed, which is a limitation of this study.

There were two samples with contradictory positive results in RT-PCR and RAT2. Because the confirmatory test was not done, it remains unclear whether it was a nonspecific reaction, interpretation error, or recording error. It is obvious though that the possibility for misinterpretation or misrecording is higher in manually reading tests instead of methods used with automatic laboratory information system.

The present study has several limitations. Due to the random nature of the peak season for these infections, the number of positive samples was unexpectedly low, especially for RSV and SARS-CoV-2 when RAT2 was evaluated. For the exact indication of the clinical performance of the tests, future studies with higher sample volume and preferably with higher incidence should be performed. Based on the RAT and RT-PCR results, the prevalence of these infections was low in our area between November 2023 and March 2024. The performance of RATs is usually improved with high prevalence (1), which may somewhat underestimate the potential of RATs in our results. Furthermore, the confirmatory tests were not executed. Confirmation of RAT result was not possible because the comparison of the RT-PCR and RAT results was not done immediately after sampling and analysis. However, the sampling and testing were performed to the best of our abilities as in routine clinical use. The samples for RT-PCR were taken immediately after RAT sampling representing the same stage of infection. RATs are used in POC testing and are done without the need for transportation in transport media. In contrast, RT-PCRs are mainly done in the laboratory when sample transporting in liquid media is required.

### Conclusions

The use of RATs might lower the costs and speed up the diagnostics when correctly used. However, because the sensitivity of the RATs remains low, their clinical use should be locally validated and risk-assessed by both laboratory and clinical infectious diseases control professionals. The negative result of RAT should be confirmed with NAAT, especially when symptomatic patients with relatively recent symptoms are admitted to a general hospital department or the use of antiviral treatment is considered.

## MATERIALS AND METHODS

### Study settings and patient selection

The study was performed at the EDs of two central hospitals, Kemi and Rovaniemi, in the area of Wellbeing Services County of Lapland, Finland. In the study, a total of 626 patients with acute respiratory symptoms (sore throat, fever, cough, running nose) who were admitted to the ED were recruited between October 2023 and March 2024. In addition to the test results, the reported duration of the symptoms at the time of sampling was collected. The specific period for RAT1 study was from the end of October 2023 to the end of January 2024 and for RAT2 study from the beginning of January 2024 to mid-March 2024.

### Sample collection and RAT and RT-PCR analysis

For RAT, the nasopharyngeal swabs from nostril 1 were first collected and analyzed immediately after sampling as per manufacturer’s instructions. Nursing personnel were pretrained to perform the analysis by the vendor. The test results were read and recorded as instructed. For RT-PCR test, the nasopharyngeal swabs from nostril 2 were collected into universal transport media (Copan, Murrieta, CA, USA) and delivered to the local laboratory as part of the routine diagnostic RT-PCR procedure. Xpert Xpress SARS-CoV-2/Flu/RSV (Cepheid, Sunnyvale, CA, USA) or NeuMoDx Flu A-B/RSV/SARS-CoV-2 (QIAGEN, Hilden, Germany) RT-PCR analysis was performed, and the results were interpreted positive or negative according to the manufacturer’s instructions. The Ct values of each RT-PCR results were collected. For Xpert Xpress, the cutoff for positivity was Ct 45, and for NeuMoDx the cutoff Ct was 37 for all the analytes.

### Data analysis

Sensitivity, specificity, positive predictive value, and negative predictive value in samples with Ct values <25, <30, and all RT-PCR positives were calculated using MedCalc Software Ltd (Ostend, Belgium). RAT1 SARS-CoV-2, Inf A, and RSV results and RAT2 InfA and RSV results were analyzed independently. The overall incidence of Inf B was low in the study period, and for the RAT2 study, SARS-CoV-2 incidence was low, which were omitted from statistical analysis. The effects of symptom duration for RAT positivity or negativity in the RT-PCR positive samples were calculated with Student’s *t* test using Analyse-IT Software (Microsoft, Redmond, WA, USA).
